# An 8.5 m long ammonite drag mark from the Upper Jurassic Solnhofen Lithographic Limestones, Germany

**DOI:** 10.1371/journal.pone.0175426

**Published:** 2017-05-10

**Authors:** Dean R. Lomax, Peter L. Falkingham, Günter Schweigert, Alejandro P. Jiménez

**Affiliations:** 1 School of Earth and Environmental Sciences, The University of Manchester, Manchester, United Kingdom; 2 Liverpool John Moores University, School of Natural Sciences and Psychology, Liverpool, United Kingdom; 3 Staatliches Museum für Naturkunde, Stuttgart, Germany; 4 CosmoCaixa Museum, Barcelona, Spain; Naturhistoriska riksmuseet, SWEDEN

## Abstract

Trackways and tracemakers preserved together in the fossil record are rare. However, the co-occurrence of a drag mark, together with the dead animal that produced it, is exceptional. Here, we describe an 8.5 m long ammonite drag mark complete with the preserved ammonite shell (*Subplanites rueppellianus*) at its end. Previously recorded examples preserve ammonites with drag marks of < 1 m. The specimen was recovered from a quarry near Solnhofen, southern Germany. The drag mark consists of continuous parallel ridges and furrows produced by the ribs of the ammonite shell as it drifted just above the sediment surface, and does not reflect behaviour of the living animal.

## Introduction

The Upper Jurassic Lithographic Limestones of Solnhofen and surrounding areas in southern Germany are renowned for exceptionally well preserved fossils. Various types of ichnofossil have been described from these Solnhofen-type limestones, and some document the trace and tracemaker together. Recorded examples include arthropods such as limulids and crustaceans [1], bivalves and snails [2,3], and fish [4], which have all been found at the end of their traces. These traces are known as mortichnia and record the last movements of an animal before death [1,5]. The longest mortichnion described to date is a 9.7 m long horseshoe crab trackway [6].

Other examples where an animal and its trace are preserved together include arthropod moulting traces [7], arm crawling crinoids [8], animals inside burrows (e.g. [9,10]), and animals found atop nests (e.g. [11,12]). Such fossils capture a specific moment in time, which is important in understanding and interpreting different behaviours in the fossil record [13].

**Fig 1:**
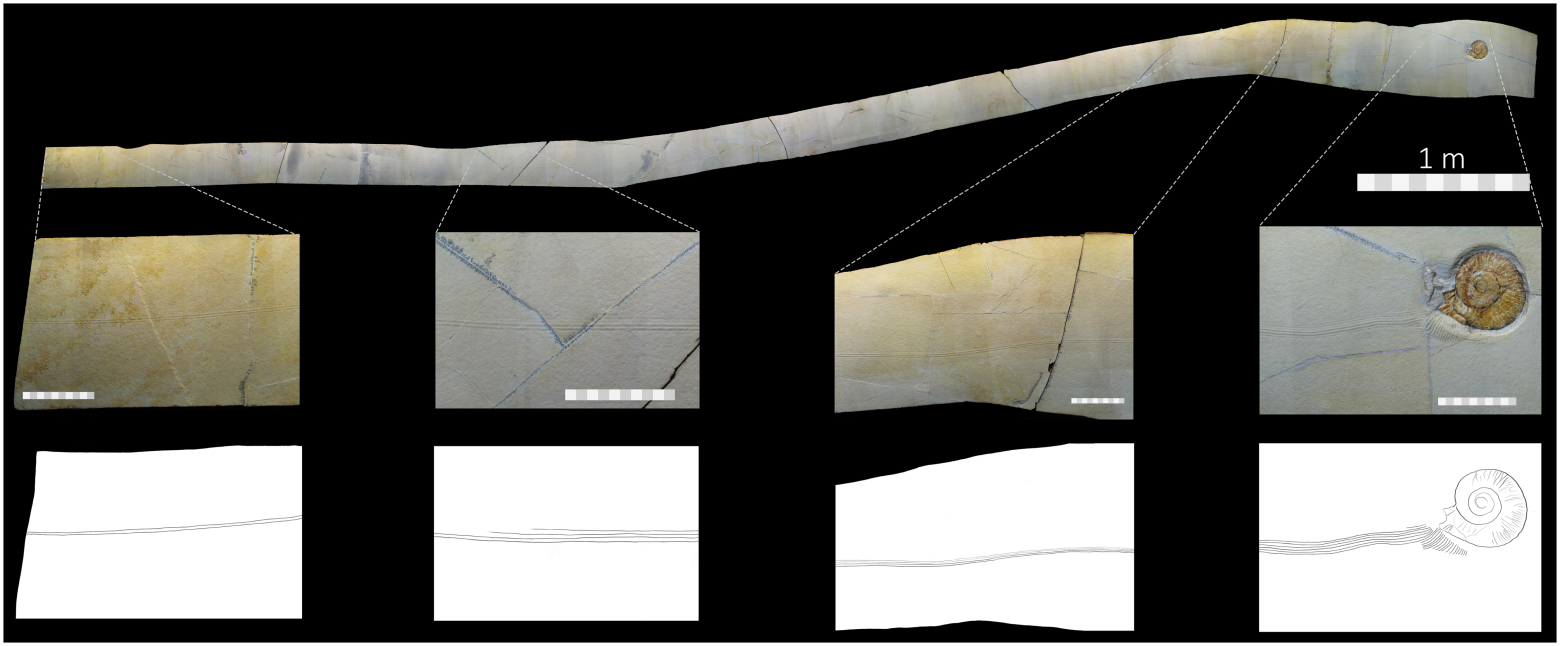
MCFO 0492, the entire drag mark created by the drifting shell of a dead ammonite (*Subplanites rueppellianus*), with close-up of several portions. A. The first portion of the drag mark clearly showing two prominent ridges. B. Drag mark showing two prominent ridges with additional faint ridges. C. Drag mark showing four prominent ridges and a gentle curve. D. Drag mark showing numerous prominent ridges, along with the ammonite. Large scale measures 1 m. Small scales measure 10 cm.

**Fig 2:**
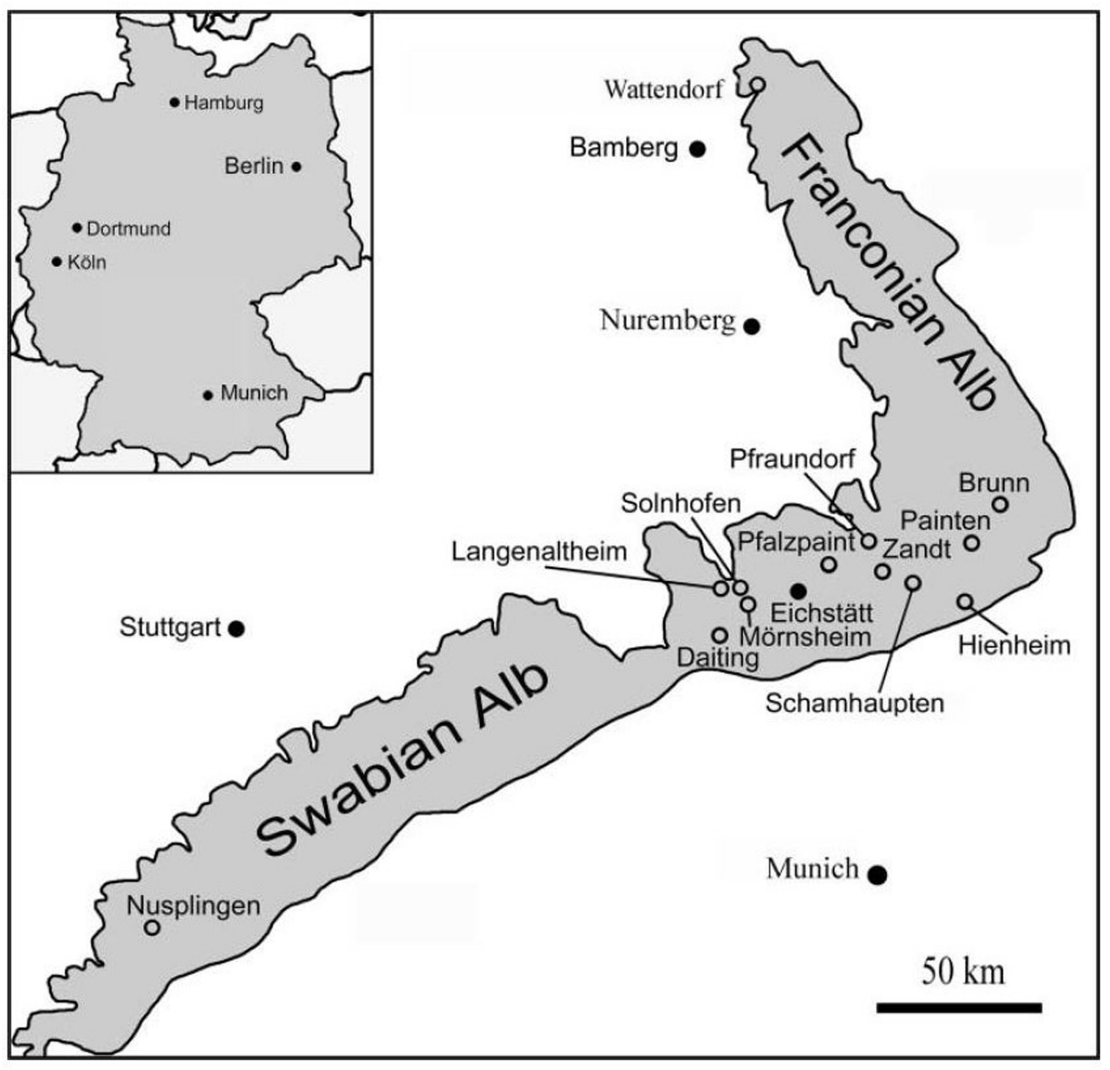
Various Plattenkalk localities of the Franconian and Swabian Alb. Note the location of Langenaltheim, near Solnhofen, the probable location of MCFO 0492. Reprinted from [35] under a CC BY license, with permission from Neues Jahrbuch für Geologie und Paläontologie, Abhandlungen, original copyright 2007.

Dead animals may also leave surface structures behind, but it is rare to find them preserved together. These structures, although in association with a body do not represent behaviour and therefore are better considered as sole marks, drag marks, or other non-biogenic structures, rather than as trace fossils (see [14]). Simple drag marks have been reported from the Solnhofen limestones, including those caused by jellyfish, driftwood, and some ammonite drag and roll marks [1–3]. Here, we describe an 8.5 m long drag mark created by the shell of a dead ammonite that is preserved at the end of its mark (MCFO 0492, in the permanent collections of the CosmoCaixa Museum, Barcelona, Spain) (Fig 1). The studied specimen was collected in the late 1990s, probably from a quarry in the Langenaltheim Haardt district, near the village of Solnhofen, Bavaria, Germany and prepared in 1998 (Fig 2). The counterpart of the studied specimen exists but it is held in a private collection. The ammonite is identified as *Subplanites rueppellianus* (Quenstedt, 1888), the index fossil of the *rueppellianus* biohorizon of the Upper Jurassic (Tithonian) Hybonotum ammonite Zone [15].

No permits were required for the described study, which complied with all relevant regulations.

## Ammonite tool marks

Ammonite shells are among the most common fossils in the Upper Jurassic Solnhofen Lithographic Limestones. However, they have been rarely in the focus of palaeontological research due to their strongly compressed, unattractive preservation [16]. Recently, it became evident that ammonites provide an important tool for high-resolution biostratigraphy when comparing the ages of the various Solnhofen limestone localities [15,17].

Several ammonite tool marks have been described from the Solnhofen limestones. As reviewed by Maeda and Seilacher [18], the first ammonite touch marks (made by the ammonite shell briefly touching the sediment surface) were initially misidentified as trace fossils produced by vertebrates; such as claw scratches, or ripple effects caused by fish swimming just above the sediment surface. Other ammonite touch marks have been reported from the Jurassic of the Champagnole region, France [19] and from the Upper Cretaceous of the Western Interior Seaway, USA [20]. Marks produced by rolling ammonite shells have also been described. The first description of several ammonite roll marks was by Abel [21], however, Abel misidentified these as trackways produced by turtles and coelacanths. Similar roll marks have been described from Slovenia [22]. Some marks have been produced by bouncing ammonites [1,23], where a rolling mark is punctuated by periodic gaps where the shell has hit the aperture while rolling.

The first report of an ammonite body fossil and drag mark was given by Rothpletz [24], who described a specimen from the Solnhofen limestone consisting of a short mark and fragmentary shell. Since then, similar drag marks from the Solnhofen limestones have been documented by Trusheim [25], Kolb [26], Seilacher [23], Barthel [27], Viohl [28], and Keupp and Schweigert [29]. The latter three are represented by relatively complete ammonites, but with drag marks all less than 1 m in length. Similarly, another specimen that has not been described in the literature (SMF XXX 838a+b, Senckenberg Museum Frankfurt, Germany) comprises a drag mark of less than 2 m, with the ammonite preserved. However, for the entire length of this specimen, the drifting ammonite shell has been dragged through a mass of algal mats.

## Description

**Fig 3:**
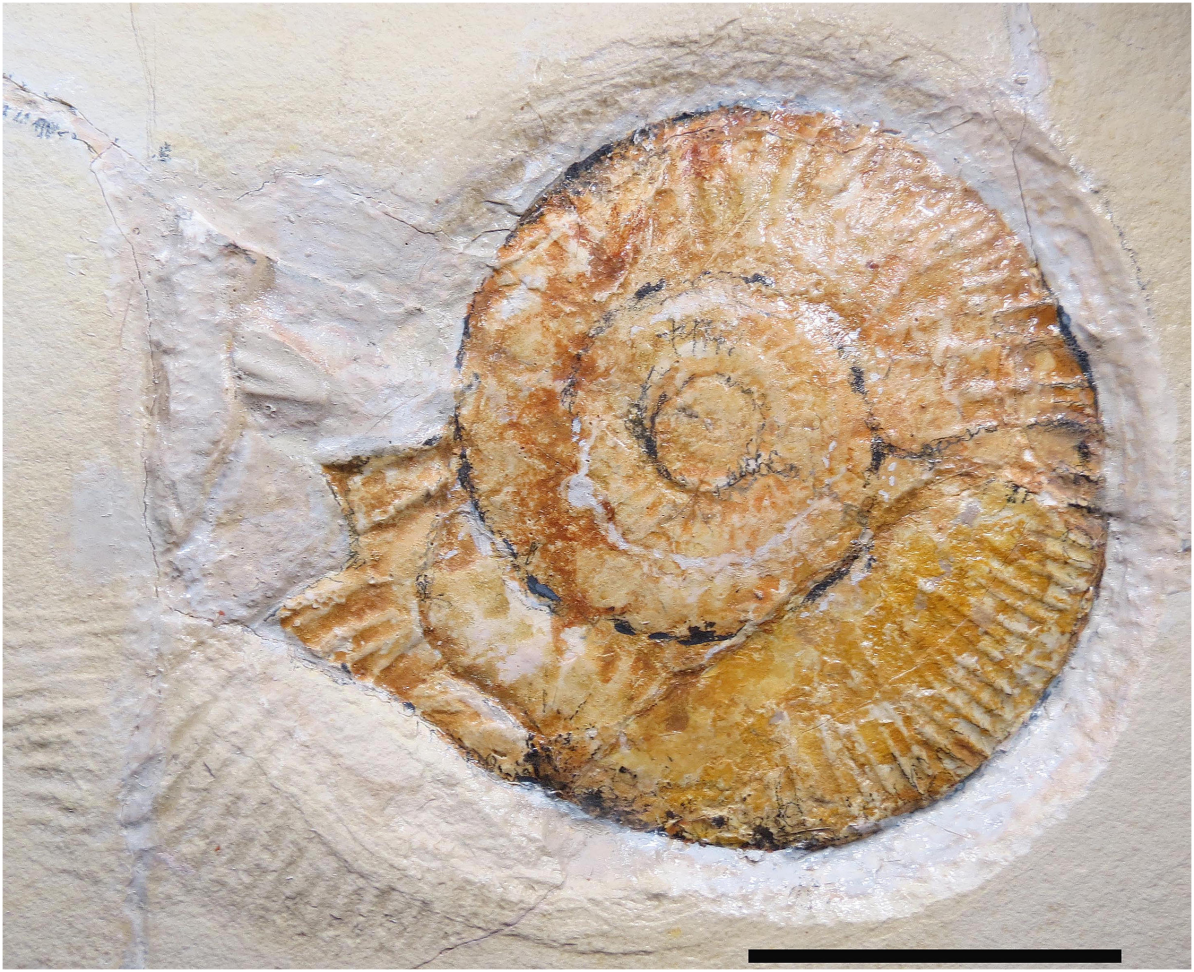
The ammonite *Subplanites rueppellianus*, the producer of the drag mark (MCFO 0492). Note the touch down mark which changes the orientation (and number) of the ridges in the substrate, anteroventral to the ammonite. Scale measures 5 cm.

The perisphinctid ammonite *Subplanites* is common from the early Tithonian Solnhofen limestones [29]. Perisphinctids are well-known for roll and drag marks [1]. The preserved ammonite of MCFO 0492 measures 114 x 101 mm, although it may have been slightly larger (Fig 3). It is somewhat poorly preserved and a crack runs anteroposteriorly through the ammonite, which has been restored. Another fragment of the lateroposterior portion has also been restored. The damage is probably due to extraction of the specimen when collected. Due to the small size and lack of a lappeted aperture, the ammonite is interpreted as a sub-adult male specimen. Since there is no aptychus (lower Jaw) in the body-chamber, the shell must be from a dead ammonite, where the soft parts including the calcified aptychus have been lost during decay.

The well-preserved drag mark of MCFO 0492 was extracted in numerous pieces, prepared, and pieced together (Fig 1). The total length, measured along the central axis of the preserved drag mark, is 8.5 m. A digital model (https://doi.org/10.6084/m9.figshare.4479734) and movie (https://doi.org/10.6084/m9.figshare.4502807) of the complete drag mark was generated using photogrammetry [30] from 645 photographs (12mp, Canon PowerShot SX40 HS). The point at which the ammonite first contacted the substrate is not preserved, and thus the drag is incomplete with no indication of how much is missing. We interpret MCFO 0492 as being a drag mark on the primary surface of the ancient sea floor because the ammonite shell, which was probably buoyant with the aid of decaying gases, could only have affected the uppermost layer of the carbonate mud. This is in accordance with the ecdysichnia described by Vallon et al. [7], but in contrast to most trace fossils described by Seilacher [5] who regarded, especially mortichnia, as undertracks.

The drag mark is comprised of a series of ridges and furrows made by the ribs of the ammonite being dragged along the substrate (Fig 3). The drag mark is largely straight, but with minor changes in lateral direction. The preserved start begins with two prominent ridges, with a single furrow. Here, the mark width measures 5.7 mm. From this point, the drag mark width was measured at approximately every 50 cm (Table 1). At one metre, additional ridges created by the ribs of the ammonite appear in the substrate, but they are faint and poorly preserved. Noticeably, at 1.7 m, an additional three ridges are present but disappear again.

**Table 1:** Measurements of the drag mark width of MCFO 0492, including the number of ridges present. Measurements marked with an * are estimated. Estimates were made when the total number of ridges were unclear.

Measurement number	Number of ridges present	Comments	Measurement along drag mark length (cm)	Width of drag mark (in mm)
**1**	2	Preserved start of drag mark	0	5.7
**2**	2		50	5.5
**3**	2	See 3a	100	5.5
**3a**	?4	Second measurement including faint ridges	100	*11.4
**4**	2	See 4a	150	5.6
**4a**	?5/6	Second measurement including faint ridges	150	*17.5
**5**	5		200	14.8
**6**	5	Includes very faint ridges	250	13
**7**	4	Four prominent ridges	300	11.9
**8**	4		350	12.2
**9**	4	Some of the ridges are faint	400	12.7
**10**	4		450	11.8
**11**	4	Four ridges present, but the mark is wider	500	14.5
**12**	4		550	13
**13**	4		600	11.7
**14**	5		650	16
**15**	5		700	14.3
**16**	4	Very prominent ridges	750	12.6
**17**	5	"	800	16
**18**	6	"	820	19
**19**	11	Last measurement taken anterior to the ammonite	845	34.8
**20**	18	Measurement of ridges in touch down mark lying anteroventral to the ammonite	850	63.2

Four ridges appear consistently from around 2 m (Fig 1), until about 6.5 m, where five prominent ridges appear. At approximately 7.5 m, only four prominent ridges can be seen, but beyond this point the drag mark preserves five very prominent ridges. It is not until the drag mark is nearly terminating, at 30 cm anterior to the ammonite, where six ridges are present and prominent. At 3 cm from the ammonite, the number of ridges increases to 11, showing that more of the ammonite is clearly in contact with the substrate (Fig 3). Here, the orientation of the ridges turns from being parallel to the long axis of the specimen to almost perpendicular to it, and increase in number to 18. Here, the ridges and furrows in the substrate mirror the spacing of the ammonite ribs that are well preserved, indicative of a touch down mark (Fig 3).

### Interpretation and review

Seilacher [1] stated that non-living objects, such as the shells of dead ammonites, may be transported along the substrate by waves, winds and currents, which would result in tool marks that can be easily mistaken for animal traces. The first interpretation of an ammonite drag mark was given by Trusheim [25], although he regarded the mark as a trace fossil created by an ammonite crawling along the seafloor. Kolb [26,31] was the first person to interpret an ammonite drag mark as the result of an ammonite swaying along the seafloor, leaving the rib impressions of the ammonite shell. He also noted lateral structures and interpreted them as imprints left by the tentacles of the dead ammonite. Seilacher [23] provided an interpretation of how different ammonite marks, including drag, roll, bounce and swaying marks, came to existence by waterlogged ammonite shells. Some modes of preservation were illustrated by Seilacher ([1] plate 57). Such scenarios have been simulated by rolling replica ammonites on carbonate mud and clay [1,3,27,32].

Seilacher [1] noted that ammonite roll marks were probably driven by turbidity currents and that some marks were likely caused by ammonites that retained some buoyancy. Kolb [26] suggested that these marks were probably produced in very shallow water. Although MCFO 0492 is exceptionally long but still incomplete, the preserved total length suggests it must have been created by a very calm but constant current, otherwise the ammonite shell would have started rolling. The Solnhofen limestones are considered to have formed in relatively shallow water (20 to 60 m) in a subtropical, probably semi-arid zone [3,33,34]. The seafloor itself was not as hostile as previously thought since there are numerous examples of life such as feeding traces around fish carcasses or moulting traces of lobsters [7]. It is also conceivable that the drag mark formed on a gentle palaeo-slope and the ammonite was moved via gravity (sinking), however, a lack of precise locality data leaves this assertion unsupported.

The number of ridges, and thus the width of the drag mark, differs throughout its length. When more ridges are present, we infer that more of the ammonite must have been in contact with the substrate (Table 1). This suggests that the orientation of the ammonite shell changed subtly as it drifted, with more or fewer ribs of the shell in contact with the substrate as it rotated.

Positioned anteroventral to the ammonite are 18 ridges. The transition of the ridges in the substrate from parallel to almost perpendicular to the longitudinal axis of the drag mark suggests that, before the ammonite came to rest on the substrate, the shell touched the substrate and rotated slightly (Fig 3).

## Conclusions

This exceptionally long fossil was produced by an ammonite shell post-mortem. The shell must have been partially buoyant, firstly because only a small portion of the shell contacts the substrate over the length of the mark, and secondly to be moved by a current that was gentle enough not to disturb the surrounding sediment. It is likely that the ammonite was losing buoyancy over the length of the drag mark, which resulted in eventual loss of all buoyancy and the ammonite falling on its side.

The drag mark of the studied specimen does not represent a mortichnion because it was not created by the animal when alive. Rather, this structure should more correctly be considered a tool mark. As such, behaviour must not be inferred from the drag mark of specimens such as MCFO 0492, and they have to be interpreted as non-biogenic structures produced by physical means [14]. MCFO 0492 represents the hitherto longest fossil drag mark created by a dead animal, complete with the animal preserved at the end.
